# Informal caregiver burden in dialysis care and how it relates to patients’ health-related quality of life and symptoms

**DOI:** 10.1093/ckj/sfae300

**Published:** 2024-10-07

**Authors:** Esmee Driehuis, Roemer J Janse, Anneke J Roeterdink, Wanda S Konijn, Thomas S van Lieshout, Theodôr J F M Vogels, Namiko A Goto, Marjolein I Broese van Groenou, Friedo W Dekker, Brigit C van Jaarsveld, Alferso C Abrahams, DOMESTICO Study Group, N Vrielink, P Leurs, G B W Pronk, J E M Arkenbout, A M Schrander, M Gijsman, N Kats, B van Nimwegen, T T Cnossen, D A Ventura da Silva, M Jellema, B C van Jaarsveld, A Sanders, J Lips, E Lormans, C van Aalst, M A G J ten Dam, E Ponse, C J A M Konings, M J de Ruiter, A Maalderink-Kornegoor, A van Eck van der Sluijs, C van den Berge, A Lips, J D Snoep, D A Ventura da Silva, M Jellema, B C van Jaarsveld, M Frankx, C R Susanto, A H Boonstra, J Mulderij, M Gijsendorffer, Y M Vermeeren, W de Ruijter, D H T IJpelaar, N H Hommes, L H M van Vliet, M van Buren, H P M Selten, J van der Horst, S H A Diepeveen, C van Hees, M Smets, T Cornelis, F Unal, K Bex, S Boorsma, B P Hoekstra, A M van Alphen, T J F M Vogels, A Kuijper, C H Beerenhout, A Hoogsteen, L Bierma, M Grubben, I Hulsebosch, E L Penne, L Zwiers, E M Wisse, A Sanders, P Dubbelman, R Smulders, C W H de Fijter, G van den Bosch, W A G van der Meijden, P F C Groeneweg-Peeters, E A J Paauwe, S J Huisman, R Krake, I Vogel, J C Verhave, N Janssen, G van Kempen, H Rouwhorst, I H H T Klein, E M Veldhuizen, W J M Bos, E Verweij, R Zekhuis, I Faber, A C Abrahams, H Vestjens, N van Daal, A J Luik, M Geelhoedt, D Jansen, A G Weenink

**Affiliations:** Department of Nephrology and Hypertension, University Medical Center Utrecht, Utrecht, The Netherlands; Department of Nephrology, Amsterdam UMC location Vrije Universiteit Amsterdam, Amsterdam, The Netherlands; Department of Clinical Epidemiology, Leiden University Medical Center, Leiden, The Netherlands; Department of Nephrology and Hypertension, University Medical Center Utrecht, Utrecht, The Netherlands; Department of Nephrology, Amsterdam UMC location Vrije Universiteit Amsterdam, Amsterdam, The Netherlands; Dutch Kidney Patients Association (NVN), Bussum, The Netherlands; Department of Nephrology, Amsterdam UMC location Vrije Universiteit Amsterdam, Amsterdam, The Netherlands; Department of Internal Medicine, Northwest Clinics, Alkmaar, The Netherlands; Dialysis Center Maxima, Maxima Medical Center, Veldhoven, The Netherlands; Department of Geriatric Medicine, Jeroen Bosch Hospital, ‘s Hertogenbosch, The Netherlands; Department of Sociology, Faculty of Social Sciences, Vrije Universiteit Amsterdam, Amsterdam, The Netherlands; Department of Clinical Epidemiology, Leiden University Medical Center, Leiden, The Netherlands; Department of Nephrology, Amsterdam UMC location Vrije Universiteit Amsterdam, Amsterdam, The Netherlands; Nephrocare Diapriva Dialysis Center, Amsterdam, The Netherlands; Amsterdam Cardiovascular Sciences, Diabetes and Metabolism, Amsterdam, The Netherlands; Department of Nephrology and Hypertension, University Medical Center Utrecht, Utrecht, The Netherlands

**Keywords:** caregiver burden, dialysis, health-related quality of life, kidney failure, symptom burden

## Abstract

**Background:**

Informal caregivers play a crucial role in dialysis care but may experience significant burden, potentially affecting both caregiver and patient outcomes. Research on caregiver burden and health-related quality of life (HRQoL) and the relation to patient-reported outcomes (PROs) is lacking. Therefore, we aimed to (i) describe informal caregivers’ experienced burden and HRQoL and (ii) investigate how these are related to dialysis patients’ HRQoL and symptoms.

**Methods:**

We conducted a cross-sectional study at dialysis initiation with 202 adult informal caregiver–dialysis patient dyads. Caregiver burden was measured with the Self-Perceived Pressure from Informal Care (SPPIC) questionnaire, HRQoL with the 12-item Short Form Health Survey (SF-12), and symptom number and burden with the Dialysis Symptom Index (DSI). Data were analysed using linear and logistic ordinal regression.

**Results:**

Around 38% of caregivers experienced moderate to high burden. Patients’ lower mental HRQoL [adjusted odds ratio (aOR) = 0.95, 95% confidence interval (CI) 0.92; 0.99], higher symptom number (aOR = 1.07, 95% CI 1.02; 1.12) and higher symptom burden (aOR = 1.03, 95% CI 1.01; 1.04) were associated with greater odds of higher caregiver burden. Patients’ lower mental HRQoL (β = 0.30, 95% CI 0.15; 0.46), higher symptom number (β = –0.55, 95% CI –0.78; –0.31) and higher symptom burden (β = –0.17, 95% CI –0.25; –0.10) were also associated with a lower mental HRQoL in caregivers.

**Conclusion:**

We show that a third of caregivers feel moderate to high burden and that caregiver burden is associated with patients’ mental HRQoL and symptoms. These findings highlight the importance of recognizing informal caregivers and the nature of their burden.

## Graphical Abstract

**Graphical Abstract:**
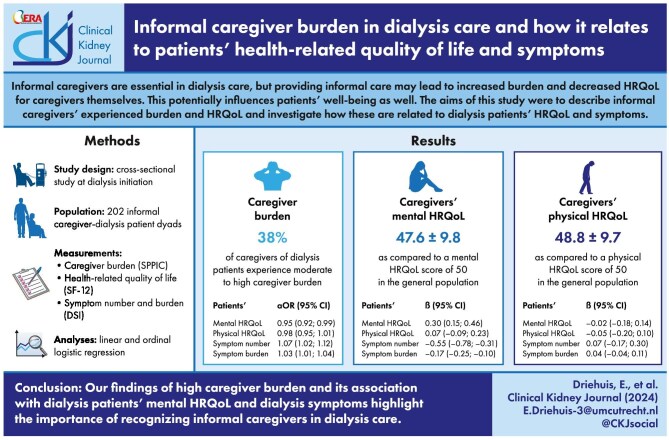

KEY LEARNING POINTS **What was known:**

- Starting dialysis has a major impact on both patients’ lives and their informal caregivers’ lives; dialysis patients often experience impaired health-related quality of life (HRQoL) and high symptom burden, relying heavily on their informal caregivers.
- Providing informal care for someone undergoing dialysis may lead to significant lifestyle changes, increased burden and decreased HRQoL for caregivers themselves; this potentially influences patients’ wellbeing as well.
- Despite the essential role of informal caregivers, there is a lack of studies formally investigating caregiver burden and HRQoL, and how these relate to patient-reported outcomes (PROs) (such as HRQoL and symptom burden), necessitating dyadic (i.e. paired) research.

**This study adds:**

- At dialysis initiation, 38% of informal caregivers of dialysis patients experience moderate to high caregiver burden; informal caregivers exhibit slightly lower mental and physical HRQoL compared with the general population.
- Lower mental HRQoL and more (severe) symptoms in dialysis patients were associated with higher caregiver burden and lower mental HRQoL in their informal caregivers.
- No associations between PROs and informal caregivers’ physical HRQoL were found; it thus might be that outcomes related to caregivers’ physical health appear to be less impacted by informal caregiving.

**Potential impact:**

- It is important to consider and support patients and their informal caregivers together in dialysis care, as they are interrelated and the kidney disease and its treatment impact many aspects of both their lives.
- Healthcare providers should recognize the vital role of informal caregivers in dialysis care and the nature of their burden.
- Future research should further elucidate the burden of caregiving and how to alleviate this (e.g. through qualitative research); in addition, to distinguish which factors are important for whom in the dyad and to alleviate the burden of kidney disease, longitudinal studies investigating causality and bidirectional effects are needed.

## INTRODUCTION

Dialysis is a kidney replacement therapy (KRT) option for patients with kidney failure, which has a major impact on patients’ daily lives, encompassing frequent dialysis sessions, regular hospital visits and lifestyle restrictions [1, 2]. Previous studies found that dialysis patients experience impairments in health-related quality of life (HRQoL) and often deal with a high symptom burden [3, 4]. As a consequence of starting dialysis, patients become increasingly dependent on informal care [5].

Informal care is defined as unpaid care to a person with whom the caregiver has a personal relationship, such as a spouse or friend [6]. Informal caregivers play a crucial role in dialysis patients’ wellbeing and successful treatment [7, 8]. However, providing informal care for someone undergoing dialysis may lead to significant lifestyle changes, increased burden and decreased HRQoL for caregivers themselves [5]. In turn, this caregiver burden may also affect patients’ HRQoL [9].

Despite the essential role of informal caregivers, there is a lack of studies investigating caregiver burden and HRQoL, and how these relate to patient-reported outcomes (PROs). Healthcare providers should be aware of these possible effects, in order to ensure that decisions regarding dialysis treatment consider both patients and their caregivers. As both dialysis patients and their caregivers prioritize research outcomes relevant to their daily living and wellbeing, these topics are important to study in dyadic (i.e. paired) research [10, 11]. Therefore, we aimed to (i) describe informal caregivers’ experienced burden and HRQoL and (ii) investigate how these are related to dialysis patients’ HRQoL and symptoms.

## MATERIALS AND METHODS

### Study population and design

For this cross-sectional study, we used baseline data from the informal caregiver study of the Dutch nOcturnal and hoME dialysis Study To Improve Clinical Outcomes (DOMESTICO) [12]. This multicentre, observational cohort study aims to compare the trajectory of experiences and HRQoL in caregivers of patients starting home dialysis versus in-centre haemodialysis (HD). This study is an extension of the DOMESTICO, a nationwide cohort study in incident dialysis patients of ≥18 years [13]. Patients with an expected kidney transplantation or life expectancy <3 months were excluded. From June 2020 until December 2022, caregivers aged ≥18 years were included. Inclusion was linked to patients’ inclusion (i.e. dyadic). If the patient had multiple caregivers, the caregiver with the highest responsibility was included. This manuscript adheres to the STrengthening the Reporting of OBservational studies in Epidemiology (STROBE) guideline (Supplementary data, Table S1) [14]. The study was conducted according to the principles of the Declaration of Helsinki. Written informed consent was obtained from all participants. The informal caregiver extension study was registered at ClinicalTrials.gov (NCT05646615) and ethical approval was obtained from the Medical Research Ethics Committee of the Amsterdam University Medical Center (NL63277.029.17).

### Measurements

Sociodemographic variables were obtained through questionnaires. Education level was grouped based on the coding system for the Dutch educational system [15]. The patient's highest finished education level was grouped into three categories: low (up to ‘low-level secondary education’), medium (‘average-level secondary education’) and high (‘high-level secondary education or university degree’). Patients’ clinical variables were obtained from their electronic patient files. Comorbidity was assessed by the Charlson Comorbidity Index (CCI), which scores the presence of 17 health conditions [16]. Having kidney failure results in a score of 2.

Caregiver burden was measured with the 15-item SPPIC (‘Self-Perceived Pressure from Informal Care’) questionnaire [17]. Every item is measured on a 3-point Likert scale. For every statement, scores are dichotomized into burden (agree = 1) or no burden (neutral or disagree = 0). The sum score ranges from 0 (no burden) to 15 (severe burden) and is divided into four categories; no burden (0 points), mild burden (1–3 points), moderate burden (4–8 points) and high burden (≥9 points) [17]. Mild burden entails feelings of responsibility. Moderate burden involves additional challenges in balancing work and family duties. High burden signifies surpassing all limits due to excessive obligations, leading to conflicts in both home and work domains, declining health and encompassing all issues present in lower burden categories [17].

The 12-item Short Form Health Survey (SF-12) was used to measure HRQoL in both caregivers and patients [18]. The SF-12 gives a mental component summary (MCS) score (i.e. mental HRQoL) and a physical component summary (PCS) score (i.e. physical HRQoL). These scores range from 0 to 100, with higher scores indicating better mental and physical HRQoL. Scores are norm-based with a mean of 50 and a standard deviation of 10 standardized to the US general population.

Patients’ symptoms were measured using the Dialysis Symptom Index (DSI) [19]. This questionnaire consists of the 30 most common physical and mental symptoms in dialysis patients. Patients indicated a symptom's presence and, if present, its severity on a 5-point Likert scale ranging from 1 (‘not at all burdensome’) to 5 (‘very burdensome’). Overall symptom burden score ranges from 0 (no symptoms) to 150 (all 30 symptoms present and very burdensome), calculated by summing all severity scores.

### Statistical analyses

Descriptive analyses were performed for baseline characteristics. Associations between PROs (patients’ mental HRQoL, physical HRQoL, symptom number and symptom burden) and informal caregivers’ experienced burden, mental HRQoL and physical HRQoL were assessed using crude (univariable) and adjusted (multivariable) linear and ordinal logistic regression analyses. Adjustment for confounders was based on two models. The first model was adjusted for caregivers’ sociodemographic variables, namely age, sex and education level. The second model was further adjusted for clinical variables, namely the patients’ dialysis modality [HD vs peritoneal dialysis (PD)] and comorbidities.

Baseline characteristics are presented as numbers (percentage) for categorical variables and as mean (standard deviation) or median (interquartile range) for continuous variables depending on the distribution. A *P*-value <.05 was considered statistically significant. Missing values were assumed to be missing at random and imputed by the Multivariate Imputation by Chained Equations (MICE) algorithm using predictive mean matching with 10 imputed datasets of 50 iterations each [20]. Clinical and questionnaire data were included in the imputation model. Missing data in questionnaires were imputed at item level from which total scores were calculated [21]. The proportion of missing data across all variables ranged from 0% to 28% (Supplementary data, Table S2). For informal caregiver data, missingness was below 6% for all variables. All analyses were performed using R version 4.2.1 (R Foundation for Statistical Computing, Vienna, Austria).

### Sensitivity analyses

We performed five sensitivity analyses to further explore robustness of our results. First, the impact of multiple imputation was investigated through complete case analysis. Second, recognizing that spousal caregivers often experience higher burden due to older age, co-residency, greater caregiving responsibilities and less social support [22–24], analyses were repeated with only spousal caregivers. The third and fourth sensitivity analyses delved into the intensity of care provision, a major stressor of caregiver burden [25]. These analyses included caregivers of patients who received no professional care and caregivers of patients who received no additional informal care. Finally, we explored the impact of categorizing caregiver burden according to the SPPIC manual by conducting a sensitivity analysis in which we analysed caregiver burden linearly (i.e. with the total score of 0 through 15).

## RESULTS

### Caregiver and patient characteristics

Of the 209 caregivers included in the cohort, 202 filled in questionnaires, resulting in 202 informal caregiver–dialysis patient dyads included in this study. Median age of caregivers was 63 years, 71% were female and 78% were spousal caregivers (Table 1). Just over 15% received additional professional care and around 17% received additional informal care. For patients, median age was 69 years, 33% were female and 36% were on PD. Of all PD patients, 54% received assistance with performing PD from their informal caregiver. Of all patients, 75% had comorbidities on top of their kidney failure. Mean MCS and PCS scores of patients were 47.5 ± 9.2 and 35.8 ± 10.4, respectively, and mean symptom number and burden were 11.5 ± 5.6 and 31.7 ± 17.3, respectively.

**Table 1:** Baseline characteristics of 202 informal caregiver-dialysis patient dyads.

	Informal caregivers (*n* = 202)	Patients (*n* = 202)
Age (years), median (IQR)	63.0 (52.0, 71.0)	69.0 (58.2, 74.8)
Women, *n* (%)	144 (71.3)	67 (33.2)
Education level, *n* (%)		
Low	89 (44.3)	79 (46.5)
Middle	48 (23.9)	43 (25.3)
High	64 (31.8)	48 (28.2)
Marital status, *n* (%)		
Single	13 (6.4)	15 (8.7)
Married/living together	181 (89.6)	136 (79.1)
Divorced	4 (2.0)	6 (3.5)
Widowed	4 (2.0)	15 (8.7)
Dialysis modality, *n* (%)		
HD		130 (64.4)
PD		72 (35.6)
Primary kidney disease, *n* (%)		
Glomerular disease		31 (16.0)
Tubulointerstitial disease		19 (9.8)
Diabetes mellitus		41 (21.1)
Hypertension/renal vascular disease		54 (27.8)
Other systemic diseases affecting the kidney		5 (2.6)
Familial/hereditary nephropathies		20 (10.3)
Miscellaneous renal disorders		24 (12.4)
CCI, *n* (%)		
2 (low)^a^		46 (25.0)
3–4 (intermediate)		80 (43.5)
≥5 (severe)		58 (31.5)
Relationship with patient, *n* (%)		
Spouse	157 (77.7)	
Sister/brother (in law)	3 (1.5)	
Daughter/son (in law)	28 (13.9)	
Parent	7 (3.5)	
Friend/family/neighbour	7 (3.5)	
Living together with patient, *n* (%)	167 (82.7)	
Employed, *n* (%)	91 (45.0)	29 (18.8)
Duration of informal caregiving, *n* (%)		
Less than a month	23 (11.9)	
Less than a year	80 (41.2)	
More than a year	91 (46.9)	
Assistance with PD^b^, *n* (%)	39 (19.3)	
Additional professional care, *n* (%)	31 (16.1)	
Additional care from other informal caregivers, *n* (%)	34 (17.5)	
Self-perceived caregiver burden, *n* (%)		
No	54 (28.6)	
Low	64 (33.9)	
Moderate	37 (19.6)	
High	34 (18.0)	
HRQoL, mean (SD)		
Mental component summary (MCS) score	47.6 (9.8)	47.5 (9.2)
Physical component summary (PCS) score	48.8 (9.7)	35.8 (10.4)
DSI, mean (SD)		
Symptom number		11.5 (5.6)
Symptom burden		31.7 (17.3)

a Kidney failure results in a CCI score of 2 points.

b Of all informal caregivers of PD patients, 54.2% assisted with performing PD.

IQR, interquartile range; SD, standard deviation.

Percentages correspond to distribution of each variable excluding missing values.

### Caregiver burden

**Figure 1:**
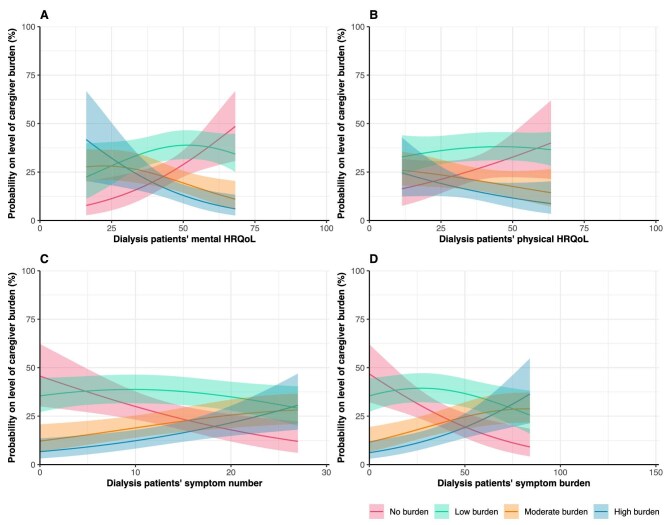
Assessment of the association between PROs and informal caregiver burden. Graphs show estimated probabilities on the level of caregiver burden with the corresponding 95% CIs. Higher scores indicate better HRQoL. Probabilities are estimated using imputed adjusted ordinal regression models.

Regarding caregiver burden, around 38% of the caregivers indicated to feel moderate to high burden [Table 1; moderate burden (19.6%) and high burden (18.0%)]. Patients’ lower mental HRQoL [adjusted odds ratio (aOR) = 0.95, 95% confidence interval (CI) 0.92; 0.99], higher symptom number (aOR = 1.07, 95% CI 1.02; 1.12) and higher symptom burden (aOR = 1.03, 95% CI 1.01; 1.04) were associated with greater odds of higher caregiver burden (Table 2, Fig. 1). In other words, having an additional five symptoms in patients was associated with 1.40 (95% CI: 1.10; 1.76) times greater odds of transitioning to the consecutive (i.e. higher) burden category. No association was found between patients’ physical HRQoL (aOR = 0.98, 95% CI 0.95; 1.01) and caregiver burden. In Fig. 1, the probabilities for each level of caregiver burden (i.e. no, low, moderate, high) are shown. Figure 1A, for example, shows that the probability of no caregiver burden increases with an increasing mental HRQoL of dialysis patients, while the probability of high caregiver burden decreases with an increasing mental HRQoL of dialysis patients.

**Table 2:** Association between PROs and informal caregiver burden and HRQoL.

	Informal caregiver outcomes		
	Caregiver burden^a^	Mental HRQoL	Physical HRQoL
PROs	OR (95% CI)	β (95% CI)	β (95% CI)
Patients’ mental HRQoL (range 0–100)			
Unadjusted	0.96 (0.93; 0.99)	0.30 (0.15; 0.46)	−0.08 (−0.25; 0.08)
Model 1^b^	0.96 (0.92; 0.99)	0.30 (0.14; 0.45)	−0.02 (−0.18; 0.15)
Model 2^c^	0.95 (0.92; 0.99)	0.30 (0.15; 0.46)	−0.02 (−0.18; 0.14)
Patients’ physical HRQoL (range 0–100)			
Unadjusted	0.98 (0.95; 1.01)	0.04 (−0.11; 0.20)	−0.04 (−0.19; 0.10)
Model 1	0.97 (0.94; 1.00)	0.06 (−0.09; 0.21)	−0.06 (−0.20; 0.08)
Model 2	0.98 (0.95; 1.01)	0.07 (−0.09; 0.23)	−0.05 (−0.20; 0.10)
Symptom number (range 0–30)			
Unadjusted	1.08 (1.03; 1.13)	−0.58 (−0.81; −0.35)	0.14 (−0.11; 0.38)
Model 1	1.07 (1.02; 1.12)	−0.54 (−0.77; −0.31)	0.07 (−0.17; 0.31)
Model 2	1.07 (1.02; 1.12)	−0.55 (−0.78; −0.31)	0.07 (−0.17; 0.30)
Symptom burden (range 0–150)			
Unadjusted	1.03 (1.01; 1.04)	−0.18 (−0.26; −0.11)	0.06 (−0.01; 0.14)
Model 1	1.03 (1.01; 1.04)	−0.17 (−0.24; −0.10)	0.04 (−0.03; 0.11)
Model 2	1.03 (1.01; 1.04)	−0.17 (−0.25; −0.10)	0.04 (−0.04; 0.11)

a ORs for transitioning to the consecutive outcome category (i.e. no burden, low burden, moderate burden and high burden).

b Adjusted for informal caregivers’ sociodemographic variables (i.e. age, sex and educational level).

c Further adjusted for patients’ dialysis modality (HD vs PD) and CCI.

### Caregivers’ mental HRQoL

**Figure 2:**
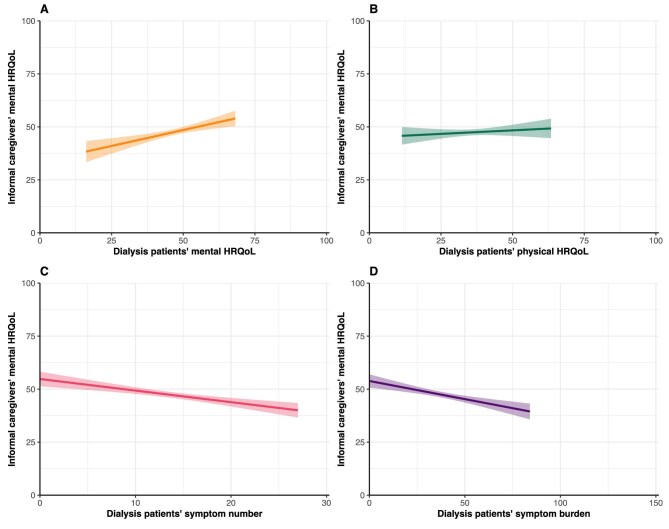
Assessment of the association between PROs and informal caregivers’ mental HRQoL. Graphs show estimated marginal means of the MCS score of the SF-12 with the corresponding 95% CIs. Higher scores indicate better HRQoL. Mean scores are estimated using imputed adjusted linear regression models.

Caregivers’ mean MCS score was 47.6 ± 9.8 (Table 1). After adjustment for confounders, patients’ lower mental HRQoL (β = 0.30, 95% CI 0.15; 0.46), higher symptom number (β = −0.55, 95% CI −0.78; −0.31) and higher symptom burden (β = −0.17, 95% CI −0.25; −0.10) were associated with a lower mental HRQoL in caregivers (Table 2, Fig. 2). To illustrate, a 5-points lower mental HRQoL in patients was associated with 1.50 points lower mental HRQoL in caregivers and having five additional symptoms in patients was associated with a 2.75-points lower mental HRQoL in caregivers. No association between patients’ physical HRQoL (β = 0.07, 95% CI −0.09; 0.23) and caregivers’ mental HRQoL was found.

### Caregivers’ physical HRQoL

**Figure 3:**
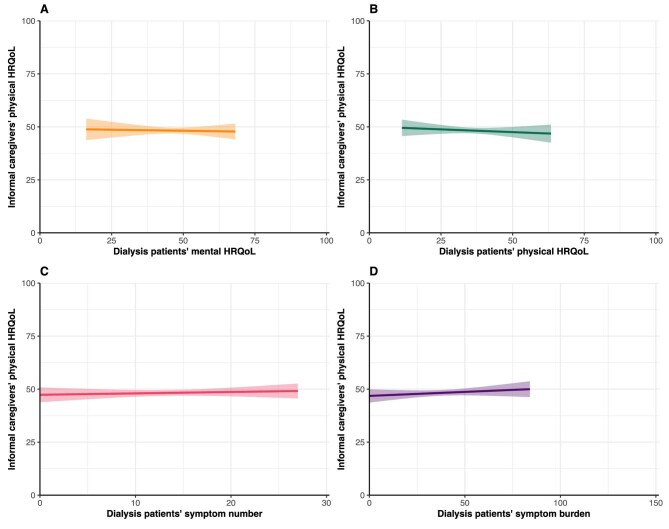
Assessment of the association between PROs and informal caregivers’ physical HRQoL. Graphs show estimated marginal means of the PCS score of the SF-12 with the corresponding 95% CIs. Higher scores indicate better HRQoL. Mean scores are estimated using imputed adjusted linear regression models.

Caregivers’ mean PCS score was 48.8 ± 9.7 (Table 1). No associations were found between patients’ mental HRQoL (β = −0.02, 95% CI −0.18; 0.14), physical HRQoL (β = −0.05, 95% CI −0.20; 0.10), symptom number (β = 0.07, 95% CI −0.17; 0.30) and symptom burden (β = 0.04, 95% CI −0.04; 0.11) and their caregivers’ physical HRQoL (Table 2, Fig. 3).

### Sensitivity analyses

Results of the first four sensitivity analyses were comparable to the main analyses (Supplementary data, Tables S3 and S4). Slightly smaller effects were found for the complete case analyses, probably due to a lower sample size. In the fifth sensitivity analysis, in which we analysed caregiver burden linearly, we encountered violation of assumptions (Supplementary data, Fig. S1). Log-transformation did not resolve this problem either.

## DISCUSSION

### Main findings

Our study shows that 38% of dialysis patients’ informal caregivers experience moderate to high caregiver burden. Patients’ lower mental HRQoL, higher symptom number and higher symptom burden were associated with greater odds of higher caregiver burden. Mental and physical HRQoL of caregivers were slightly lower than in the general population. Lower mental HRQoL, higher symptom number, and higher symptom burden in patients were associated with lower mental HRQoL of their caregivers, whereas no PROs were associated with caregivers’ physical HRQoL. Sensitivity analyses showed similar results for spouses and for caregivers with higher care intensity (i.e. without additional informal or professional care).

### Caregiver burden

A study in caregivers of dialysis patients aged ≥65 years showed that 23% of the caregivers experienced moderate to high burden at dialysis initiation [26]. Nonetheless, the authors reported this percentage increased to 38% at 6 months. We found a notably higher caregiver burden at dialysis initiation, namely 38% with moderate to high burden. As in the other study, this percentage may also increase over time. The discrepancy in burden at dialysis initiation might be due to demographic and clinical differences, such as our relatively high proportion home dialysis patients. The high burden in our study highlights the importance and magnitude of the problem, also considering that overburdening may lead to negative outcomes for both caregivers and patients. A systematic review in caregivers of dialysis patients confirmed a significant caregiver burden as well, albeit that the overall quality of included studies was poor and multiple burden scales were used [27]. To align further research and practice, we recommend use of the SPPIC questionnaire to measure caregiver burden, as this is a valuable tool for screening for different categories of overburdening. The tool provides specific insight into two target groups for policy and support: the overburdened group that is (at risk of being) adversely affected by the caregiving tasks and those who experience participation problems as a result of providing informal care [17].

Regarding the association between PROs and caregiver burden, one systematic review found that disease-related factors, such as patients’ experienced symptoms, were associated with increased caregiver burden [28]. In addition, a study in caregivers of HD patients revealed that especially treatment-related tasks contributed to high levels of caregiver burden [29]. An association between patients’ physical status and caregiver burden was also found [28, 30]. In our study, this association may have been more accurately captured by patients’ symptom number and burden rather than by patients’ physical HRQoL. Physical HRQoL does not capture the objective physical status of a patient, as coping mechanisms and other factors may lead to patients not feeling physically limited.

The similar associations for caregiver burden and caregivers’ mental HRQoL may be caused by a negative association between these caregiver outcomes [28, 31]. Still, it is useful to measure both caregiver burden and mental HRQoL. In conceptual models, such as Pearlin's caregiver stress model, these are considered distinctive concepts [25]. Moreover, empirical studies show that caregivers may be burdened while simultaneously experiencing adequate mental HRQoL [32].

### Caregivers’ HRQoL

In accordance with our results, previous research showed that caregivers’ mental and physical HRQoL were slightly poorer than in the general population and mostly comparable to caregivers of patients with other chronic diseases [27]. We found that patients’ mental HRQoL, symptom number and symptom burden were associated with caregivers’ mental HRQoL, which is in contrast to the only other study on the association between PROs and caregivers’ HRQoL. This Japanese study in 51 informal caregiver–dialysis patient dyads found no association between caregivers’ HRQoL and patients’ HRQoL and symptoms [33]. The low number of dyads, culture differences and the dichotomization of HRQoL in this study may be the cause of the disparate results. Nonetheless, our findings are consistent with Pearlin's caregiver stress model, according to which the caregiving situation (e.g. patients’ experienced symptoms) is an important stressor of caregivers’ mental wellbeing [25]. Our sensitivity analyses show that the associations with caregivers’ mental HRQoL remain in the groups with higher care intensity, which is another important stressor of Pearlin's model.

Although one may hypothesize that a patient's worse physical status may lead to more (intensive) caregiving tasks, which subsequently may lead to a worse mental HRQoL, we found no association between patients’ physical HRQoL and caregivers’ mental HRQoL. Studies in other fields do show an association between patients’ physical impairments and lower mental HRQoL of their caregivers [34, 35]. This discrepancy may be due to dialysis patients do not having very pronounced physical limitations, as opposed to for example patients with muscular dystrophy. In addition, as aforementioned, we did not objectively measure physical status or impairments, but physical HRQoL.

Regarding physical HRQoL of caregivers, no associations with PROs were found. Although previous literature shows that providing informal care for dialysis patients may lead to worse physical wellbeing [36], our results indicate that this was not be associated with patients’ HRQoL and symptoms. An explanation for our findings might be that outcomes related to caregivers’ physical health, compared with mental health, appear to be less impacted by informal caregiving, as also suggested by several studies [37]. This may be explained by the fact that an individual's capacity to provide informal care depends on their ability to maintain their physical health and function (i.e. people with poor physical health are often not able to provide informal care) [37].

### Strengths and limitations

To our knowledge, this is the first study to explore caregiver burden and HRQoL and how this relates to dialysis patients’ PROs in detail. A major strength of our study is the dyadic data, which allowed us to investigate the association between PROs and informal caregiver-reported outcomes in every caregiver–patient dyad. In addition, our data source was granular and homogeneous. Our sample size is larger than in other studies and includes both caregivers of patients on HD and PD. Lastly, robustness of the results was examined through comprehensive sensitivity analyses.

A limitation is the cross-sectional nature of this study, implying that no causal statements can be made. Likely, there is a bidirectional bond between dialysis patients and their informal caregivers, as they are interrelated [38]. In addition, questionnaires were filled in within 3 months after dialysis initiation, but the exact moment varied by and within dyads. The timing of questionnaire completion may have affected our results, as previous research indicates that patients’ HRQoL improved right after starting dialysis while caregiver HRQoL worsened (although it improved again by 3 months) [39]. Lastly, as our cohort consisted mainly of spousal caregivers, generalizability to other types of caregivers is limited. Subgroup analyses to explore generalizability were not possible due to a limited number of caregivers per subgroup.

### Implications for research and practice

Our findings of high burden and decreased HRQoL among informal caregivers and its association with patients’ mental HRQoL and symptoms emphasize the importance of considering patients and informal caregivers together in dialysis care. For instance, besides the negative consequences for the caregivers themselves, overburdening of caregivers may mean that patients do not get the support and care they need. Our findings underline the need for a holistic approach to dialysis care, recognizing the emotional and mental health challenges faced by both patients and caregivers. For example, psychological support for both patients and caregivers could be beneficial. To distinguish which factors are important for whom in the dyad and to alleviate the burden of kidney disease, future longitudinal studies investigating causality and bidirectional effects are needed. A topic of special interest herein would be the impact of dialysis modality due to the important role of informal caregivers in home dialysis. Additionally, in further elucidating and alleviating the burden of caregiving, qualitative research may play an essential role.

## CONCLUSIONS

In conclusion, we unravel the high burden of informal caregivers of dialysis patients and show that this is associated with their patients’ mental HRQoL and dialysis symptoms. Our findings highlight the importance of recognizing informal caregivers in dialysis care and the nature of their burden. A holistic approach is needed in dialysis care. The emotional and mental health challenges faced by both patients and caregivers should be considered, in which psychological support may play a key role. Additionally, further research should focus on identifying causal factors and to determine how to alleviate the burden of caregivers.

## Supplementary Material

sfae300_Supplemental_File

## Data Availability

The data that support the findings of this study are available from the corresponding author upon reasonable request and with permission of the DOMESTICO study group.
