# Supplementary material for: Informal caregiver burden in dialysis care and how it relates to patients’ health-related quality of life and symptoms
Source: Clin Kidney J. 2024 Oct 7;17(11):sfae300. doi: 10.1093/ckj/sfae300 (PMC11528300; doi:10.1093/ckj/sfae300)

Informal caregiver burden in dialysis care and how it relates to patients' health-related quality of life and symptoms

Esmee Driehuis, Roemer J Janse, Anneke J Roeterdink, Wanda S Konijn, Thomas S van Lieshout, Theodôr JFM Vogels, Namiko A Goto, Marjolein I Broese van Groenou, Friedo W Dekker, Brigit C van Jaarsveld, Alferso C Abrahams, on behalf of the DOMESTICO study group.

SUPPLEMENTARY MATERIAL

Supplementary Table 1. STROBE Statement—Checklist of items that should be included in reports of cross-sectional studies

Supplementary Table 2. Missing data of all variables in this study.

Supplementary Table 3. Four sensitivity analyses of caregiver burden: complete case analysis; only spousal caregivers; caregivers of patients who received no professional care; caregivers of patients who received no extra informal care.

Supplementary Table 4: Four sensitivity analyses of informal caregivers' mental and physical HRQoL: complete case analysis; only spousal caregivers; caregivers of patients who received no professional care; caregivers of patients who received no extra informal care.

Supplementary Figure 1. Violation of assumptions for homoscedasticity and normality for the fifth sensitivity analysis treating caregiver burden as a continuous outcome. A = patients' mental HRQoL; B = patients' physical HRQoL; C = symptom number; D = symptom burden; 1 = homoscedasticity; 2 = normality of residuals.

Supplementary Table 1. STROBE Statement—Checklist of items that should be included in reports of cross-sectional studies

	Item	Recommendation	Reported
**Title and abstract**	1	(*a*) Indicate the study's design with a commonly used term in the title or the abstract	✓
		(*b*) Provide in the abstract an informative and balanced summary of what was done and what was found	✓
**Introduction**			
Background/rationale	2	Explain the scientific background and rationale for the investigation being reported	✓
Objectives	3	State specific objectives, including any prespecified hypotheses	✓ (no prespecified hypotheses were formulated)
**Methods**			
Study design	4	Present key elements of study design early in the paper	✓
Setting	5	Describe the setting, locations, and relevant dates, including periods of recruitment, exposure, follow-up, and data collection	✓
Participants	6	(*a*) Give the eligibility criteria, and the sources and methods of selection of participants	✓
Variables	7	Clearly define all outcomes, exposures, predictors, potential confounders, and effect modifiers. Give diagnostic criteria, if applicable	✓
Data sources/ measurement	8	For each variable of interest, give sources of data and details of methods of assessment (measurement). Describe comparability of assessment methods if there is more than one group	✓
Bias	9	Describe any efforts to address potential sources of bias	✓
Study size	10	Explain how the study size was arrived at	not applicable: study size was calculated in the protocol of the main study
Quantitative variables	11	Explain how quantitative variables were handled in the analyses. If applicable, describe which groupings were chosen and why	✓
Statistical methods	12	(*a*) Describe all statistical methods, including those used to control for confounding	✓
		(*b*) Describe any methods used to examine subgroups and interactions	not applicable: no subgroups or interactions were examined
		(*c*) Explain how missing data were addressed	✓
		(*d*) If applicable, describe analytical methods taking account of sampling strategy	not applicable: description in the protocol of the main study
		(*e*) Describe any sensitivity analyses	✓
**Results**			
Participants	13	(a) Report numbers of individuals at each stage of study—e.g. numbers potentially eligible, examined for eligibility, confirmed eligible, included in the study, completing follow-up, and analysed	✓ (numbers (potentially) eligible not available)
		(b) Give reasons for non-participation at each stage	✓ (only available for exclusion based on non-response)
		(c) Consider use of a flow diagram	✗

Descriptive data	14	(a) Give characteristics of study participants (e.g. demographic, clinical, social) and information on exposures and potential confounders	✓
		(b) Indicate number of participants with missing data for each variable of interest	✓
Outcome data	15	Report numbers of outcome events or summary measures	✓
Main results	16	(*a*) Give unadjusted estimates and, if applicable, confounder-adjusted estimates and their precision (e.g., 95% confidence interval). Make clear which confounders were adjusted for and why they were included	✓
		(*b*) Report category boundaries when continuous variables were categorized	✓
		(*c*) If relevant, consider translating estimates of relative risk into absolute risk for a meaningful time period	not applicable
Other analyses	17	Report other analyses done—e.g. analyses of subgroups and interactions, and sensitivity analyses	✓
**Discussion**			
Key results	18	Summarise key results with reference to study objectives	✓
Limitations	19	Discuss limitations of the study, taking into account sources of potential bias or imprecision. Discuss both direction and magnitude of any potential bias	✓
Interpretation	20	Give a cautious overall interpretation of results considering objectives, limitations, multiplicity of analyses, results from similar studies, and other relevant evidence	✓
Generalisability	21	Discuss the generalisability (external validity) of the study results	✓
**Other information**			
Funding	22	Give the source of funding and the role of the funders for the present study and, if applicable, for the original study on which the present article is based	✓

Supplementary Table 2. Missing data of all variables in this study.

Variable	Percentage missing
**Informal caregivers' characteristics**	
Age	0.5%
Sex	-
Educational level	0.5%
Marital status	-
Relationship with patient	-
Living together with patient	-
Employed	-
Duration of informal caregiving	4.0%
Assistance with PD	-
Additional professional care	5.0%
Additional care from other informal caregivers	4.0%
**Patients' characteristics**	
Age	-
Sex	-
Educational level	15.8%
Marital status	14.9%
Dialysis modality	-
Primary kidney disease	4.0%
Charlson Comorbidity Index (CCI)	8.9%
Employed	23.8%
**Caregiver burden (EDIZ+)**	
Question 1	1.0%
Question 2	1.0%
Question 3	1.0%
Question 4	1.0%
Question 5	2.0%
Question 6	2.0%
Question 7	1.5%
Question 8	1.5%
Question 9	3.5%
Question 10	1.5%
Question 11	1.0%
Question 12	1.0%
Question 13	1.0%
Question 14	1.0%
Question 15	1.5%
**Informal caregivers' HRQoL (SF12)**	
Question 1	4.0%
Question 2a	4.5%
Question 2b	5.0%
Question 3a	4.0%
Question 3b	5.0%
Question 4a	4.0%
Question 4b	4.5%
Question 5	2.5%
Question 6a	5.0%
Question 6b	6.0%
Question 6c	5.5%
Question 7	3.5%
**Patients' HRQoL (SF12)**	
Question 1	23.3%
Question 2a	22.3%
Question 2b	26.2%
Question 3a	24.8%
Question 3b	23.3%
Question 4a	23.8%

Question 4b	26.2%	
Question 5	25.2%	
Question 6a	24.3%	
Question 6b	24.3%	
Question 6c	23.8%	
Question 7	23.8%	
**Dialysis symptom index (DSI)**	Yes/no	Burden
Constipation	23.8%	23.8%
Nausea	23.8%	23.8%
Vomiting	23.8%	23.8%
Diarrhoea	23.8%	23.8%
Decreased appetite	23.8%	23.8%
Muscle cramps	23.8%	23.8%
Swelling in legs	23.8%	23.8%
Shortness of breath	23.8%	23.8%
Light-headedness or dizziness	23.8%	23.8%
Restless legs	23.8%	23.8%
Numbness or tingling in feet	23.8%	23.8%
Feeling tired or lack of energy	23.8%	24.3%
Cough	23.8%	23.8%
Dry mouth	23.8%	23.8%
Bone or joint pain	23.8%	23.8%
Chest pain	24.3%	24.3%
Headache	23.3%	23.3%
Muscle soreness	22.3%	22.8%
Difficulty concentrating	23.3%	23.3%
Dry skin	22.8%	24.8%
Itching	22.8%	23.8%
Worrying	23.3%	23.8%
Feeling nervous	23.3%	23.8%
Trouble falling asleep	22.8%	23.8%
Trouble staying asleep	22.8%	24.3%
Feeling irritable	23.3%	23.3%
Feeling sad	23.3%	23.3%
Feeling anxious	23.3%	23.8%
Decreased interest in sex	27.7%	28.2%
Difficulty becoming sexually aroused	27.2%	27.7%

Supplementary Table 3. Four sensitivity analyses of caregiver burden: complete case analysis; only spousal caregivers; caregivers of patients who received no professional care; caregivers of patients who received no extra informal care.

	Caregiver burden[a]	
**Patient-reported outcomes**	*OR (95% CI)*	*n*
**Patients' mental HRQoL**		
Unadjusted		
Main results	0.96 (0.93; 0.99)	202
Complete case	0.97 (0.93; 1.00)	126
Spouses	0.96 (0.92; 1.00)	157
No professional care	0.96 (0.92; 0.99)	161
No extra informal care	0.96 (0.92; 0.99)	160
Model 1[b]		
Main results	0.96 (0.92; 0.99)	202
Complete case	0.96 (0.92; 0.99)	125
Spouses	0.96 (0.92; 1.00)	157
No professional care	0.95 (0.92; 0.99)	161
No extra informal care	0.95 (0.92; 0.99)	160
Model 2[c]		
Main results	0.95 (0.92; 0.99)	202
Complete case	0.96 (0.93; 1.00)	115
Spouses	0.96 (0.92; 0.99)	157
No professional care	0.95 (0.92; 0.99)	161
No extra informal care	0.95 (0.91; 0.99)	160
**Patients' physical HRQoL**		
Unadjusted		
Main results	0.98 (0.95; 1.01)	202
Complete case	0.97 (0.94; 1.00)	126
Spouses	0.99 (0.95; 1.02)	157
No professional care	0.98 (0.95; 1.01)	161
No extra informal care	0.97 (0.94; 1.01)	160
Model 1		
Main results	0.97 (0.94; 1.00)	202
Complete case	0.97 (0.94; 1.00)	125
Spouses	0.98 (0.94; 1.01)	157
No professional care	0.97 (0.94; 1.00)	161
No extra informal care	0.97 (0.93; 1.00)	160
Model 2		
Main results	0.98 (0.95; 1.01)	202
Complete case	0.98 (0.95; 1.01)	115
Spouses	0.98 (0.95; 1.01)	157
No professional care	0.97 (0.94; 1.01)	161
No extra informal care	0.97 (0.93; 1.00)	160
**Symptom number**		
Unadjusted		
Main results	1.08 (1.03; 1.13)	202
Complete case	1.03 (0.98; 1.09)	133
Spouses	1.07 (1.02; 1.13)	157
No professional care	1.09 (1.03; 1.15)	161
No extra informal care	1.07 (1.02; 1.13)	160
Model 1		
Main results	1.07 (1.02; 1.12)	202
Complete case	1.03 (0.98; 1.09)	132
Spouses	1.07 (1.02; 1.13)	157
No professional care	1.08 (1.03; 1.14)	161
No extra informal care	1.07 (1.01; 1.12)	160
Model 2		
Main results	1.07 (1.02; 1.12)	202
Complete case	1.03 (0.97; 1.09)	122

	Caregiver burden[a]	
**Patient-reported outcomes**	*OR (95% CI)*	*n*
Spouses	1.07 (1.02; 1.13)	157
No professional care	1.08 (1.03; 1.14)	161
No extra informal care	1.07 (1.01; 1.12)	160
**Symptom burden**		
Unadjusted		
Main results	1.03 (1.01; 1.04)	202
Complete case	1.02 (1.00; 1.03)	129
Spouses	1.03 (1.01; 1.04)	157
No professional care	1.03 (1.02; 1.05)	161
No extra informal care	1.03 (1.01; 1.04)	160
Model 1		
Main results	1.03 (1.01; 1.04)	202
Complete case	1.02 (1.00; 1.04)	128
Spouses	1.03 (1.01; 1.04)	157
No professional care	1.03 (1.01; 1.05)	161
No extra informal care	1.02 (1.01; 1.04)	160
Model 2		
Main results	1.03 (1.01; 1.04)	202
Complete case	1.02 (1.00; 1.04)	118
Spouses	1.03 (1.01; 1.04)	157
No professional care	1.03 (1.01; 1.05)	161
No extra informal care	1.02 (1.01; 1.04)	160

[a]Odds ratios for transitioning to the consecutive outcome category (i.e., no burden, low burden, moderate burden, and high burden).
[b]Adjusted for informal caregivers' sociodemographic variables (i.e., age, sex, and educational level).
[c]Further adjusted for patients' dialysis modality (haemodialysis vs. peritoneal dialysis) and Charlson Comorbidity Index (CCI).
OR, odds ratio; 95% CI, 95% confidence interval; *n*, number of dyads included in analysis; HRQoL, health-related quality of life.

Supplementary Table 4: Four sensitivity analyses of informal caregivers' mental and physical HRQoL: complete case analysis; only spousal caregivers; caregivers of patients who received no professional care; caregivers of patients who received no extra informal care.

	Informal caregivers' HRQoL		
	**Mental HRQoL**	**Physical HRQoL**	
**Patient-reported outcomes**	*β (95% CI)*	*β (95% CI)*	*n*
**Patients' mental HRQoL**			
Unadjusted			
Main results	0.30 (0.15; 0.46)	-0.08 (-0.25; 0.08)	202
Complete case	0.29 (0.09; 0.49)	-0.18 (-0.39; 0.03)	121
Spouses	0.33 (0.16; 0.49)	-0.07 (-0.26; 0.12)	157
No professional care	0.34 (0.17; 0.52)	-0.10 (-0.28; 0.07)	161
No extra informal care	0.33 (0.17; 0.49)	-0.06 (-0.25; 0.13)	160
Model 1[a]			
Main results	0.30 (0.14; 0.45)	-0.02 (-0.18; 0.15)	202
Complete case	0.32 (0.11; 0.53)	-0.05 (-0.26; 0.16)	120
Spouses	0.31 (0.14; 0.48)	0.00 (-0.19; 0.19)	157
No professional care	0.35 (0.17; 0.52)	-0.03 (-0.21; 0.15)	161
No extra informal care	0.31 (0.15; 0.47)	0.00 (-0.19; 0.18)	160
Model 2[b]			
Main results	0.30 (0.15; 0.46)	-0.02 (-0.18; 0.14)	202
Complete case	0.26 (0.03; 0.48)	-0.02 (-0.25; 0.20)	110
Spouses	0.31 (0.14; 0.48)	0.00 (-0.18; 0.18)	157
No professional care	0.35 (0.17; 0.52)	-0.03 (-0.20; 0.14)	161
No extra informal care	0.32 (0.16; 0.48)	0.00 (-0.19; 0.18)	160
**Patients' physical HRQoL**			
Unadjusted			
Main results	0.04 (-0.11; 0.20)	-0.04 (-0.19; 0.10)	202
Complete case	0.07 (-0.10; 0.24)	0.00 (-0.18; 0.18)	121
Spouses	0.03 (-0.13; 0.19)	-0.06 (-0.23; 0.10)	157
No professional care	0.09 (-0.08; 0.26)	-0.05 (-0.21; 0.12)	161
No extra informal care	0.07 (-0.09; 0.23)	0.01 (-0.16; 0.17)	160
Model 1			
Main results	0.06 (-0.09; 0.21)	-0.06 (-0.20; 0.08)	202
Complete case	0.08 (-0.09; 0.26)	-0.03 (-0.19; 0.14)	120
Spouses	0.08 (-0.09; 0.24)	-0.11 (-0.27; 0.05)	157
No professional care	0.13 (-0.04; 0.29)	-0.10 (-0.26; 0.06)	161
No extra informal care	0.11 (-0.05; 0.27)	-0.03 (-0.19; 0.13)	160
Model 2			
Main results	0.07 (-0.09; 0.23)	-0.05 (-0.20; 0.10)	202
Complete case	0.06 (-0.13; 0.24)	0.00 (-0.18; 0.19)	110
Spouses	0.08 (-0.09; 0.25)	-0.10 (-0.26; 0.06)	157
No professional care	0.13 (-0.04; 0.30)	-0.09 (-0.25; 0.07)	161
No extra informal care	0.11 (-0.05; 0.28)	-0.03 (-0.20; 0.13)	160
**Symptom number**			
Unadjusted			
Main results	-0.58 (-0.81; -0.35)	0.14 (-0.11; 0.38)	202
Complete case	-0.61 (-0.91; -0.31)	0.15 (-0.18; 0.49)	126
Spouses	-0.58 (-0.84; -0.33)	0.23 (-0.06; 0.51)	157
No professional care	-0.62 (-0.88; -0.36)	0.23 (-0.04; 0.49)	161
No extra informal care	-0.63 (-0.86; -0.39)	0.16 (-0.11; 0.42)	160
Model 1			
Main results	-0.54 (-0.77; -0.31)	0.07 (-0.17; 0.31)	202
Complete case	-0.58 (-0.88; -0.28)	0.06 (-0.26; 0.38)	125
Spouses	-0.55 (-0.80; -0.30)	0.17 (-0.10; 0.44)	157
No professional care	-0.57 (-0.83; -0.31)	0.14 (-0.11; 0.39)	161
No extra informal care	-0.58 (-0.82; -0.35)	0.12 (-0.13; 0.38)	160
Model 2			
Main results	-0.55 (-0.78; -0.31)	0.07 (-0.17; 0.30)	202

	Informal caregivers' HRQoL		
	**Mental HRQoL**	**Physical HRQoL**	
**Patient-reported outcomes**	*β (95% CI)*	*β (95% CI)*	*n*
Complete case	-0.52 (-0.84; -0.21)	0.04 (-0.30; 0.38)	116
Spouses	-0.55 (-0.81; -0.30)	0.16 (-0.11; 0.43)	157
No professional care	-0.57 (-0.82; -0.31)	0.13 (-0.12; 0.37)	161
No extra informal care	-0.59 (-0.82; -0.35)	0.13 (-0.12; 0.39)	160
**Symptom burden**			
Unadjusted			
Main results	-0.18 (-0.26; -0.11)	0.06 (-0.01; 0.14)	202
Complete case	-0.19 (-0.29; -0.09)	0.07 (-0.04; 0.17)	123
Spouses	-0.18 (-0.26; -0.10)	0.09 (0.00; 0.18)	157
No professional care	-0.20 (-0.28; -0.12)	0.08 (0.00; 0.17)	161
No extra informal care	-0.19 (-0.27; -0.12)	0.08 (-0.01; 0.16)	160
Model 1			
Main results	-0.17 (-0.24; -0.10)	0.04 (-0.03; 0.11)	202
Complete case	-0.19 (-0.29; -0.09)	0.03 (-0.08; 0.13)	122
Spouses	-0.17 (-0.25; -0.09)	0.07 (-0.02; 0.15)	157
No professional care	-0.18 (-0.27; -0.10)	0.06 (-0.02; 0.13)	161
No extra informal care	-0.18 (-0.26; -0.10)	0.06 (-0.02; 0.15)	160
Model 2			
Main results	-0.17 (-0.25; -0.10)	0.04 (-0.04; 0.11)	202
Complete case	-0.17 (-0.27; -0.07)	0.03 (-0.08; 0.14)	113
Spouses	-0.17 (-0.25; -0.09)	0.06 (-0.02; 0.15)	157
No professional care	-0.18 (-0.27; -0.10)	0.05 (-0.03; 0.13)	161
No extra informal care	-0.18 (-0.26; -0.10)	0.06 (-0.02; 0.15)	160

[a]Adjusted for informal caregivers' sociodemographic variables (i.e., age, sex, and educational level).
[b]Further adjusted for patients' dialysis modality (haemodialysis vs. peritoneal dialysis) and Charlson Comorbidity Index (CCI).
HRQoL, health-related quality of life; 95% confidence interval; *n*, number of dyads included in analysis.

Supplementary Figure 1. Violation of assumptions for homoscedasticity and normality for the fifth sensitivity analysis treating caregiver burden as a continuous outcome. A = patients' mental HRQoL; B = patients' physical HRQoL; C = symptom number; D = symptom burden; 1 = homoscedasticity; 2 = normality of residuals.

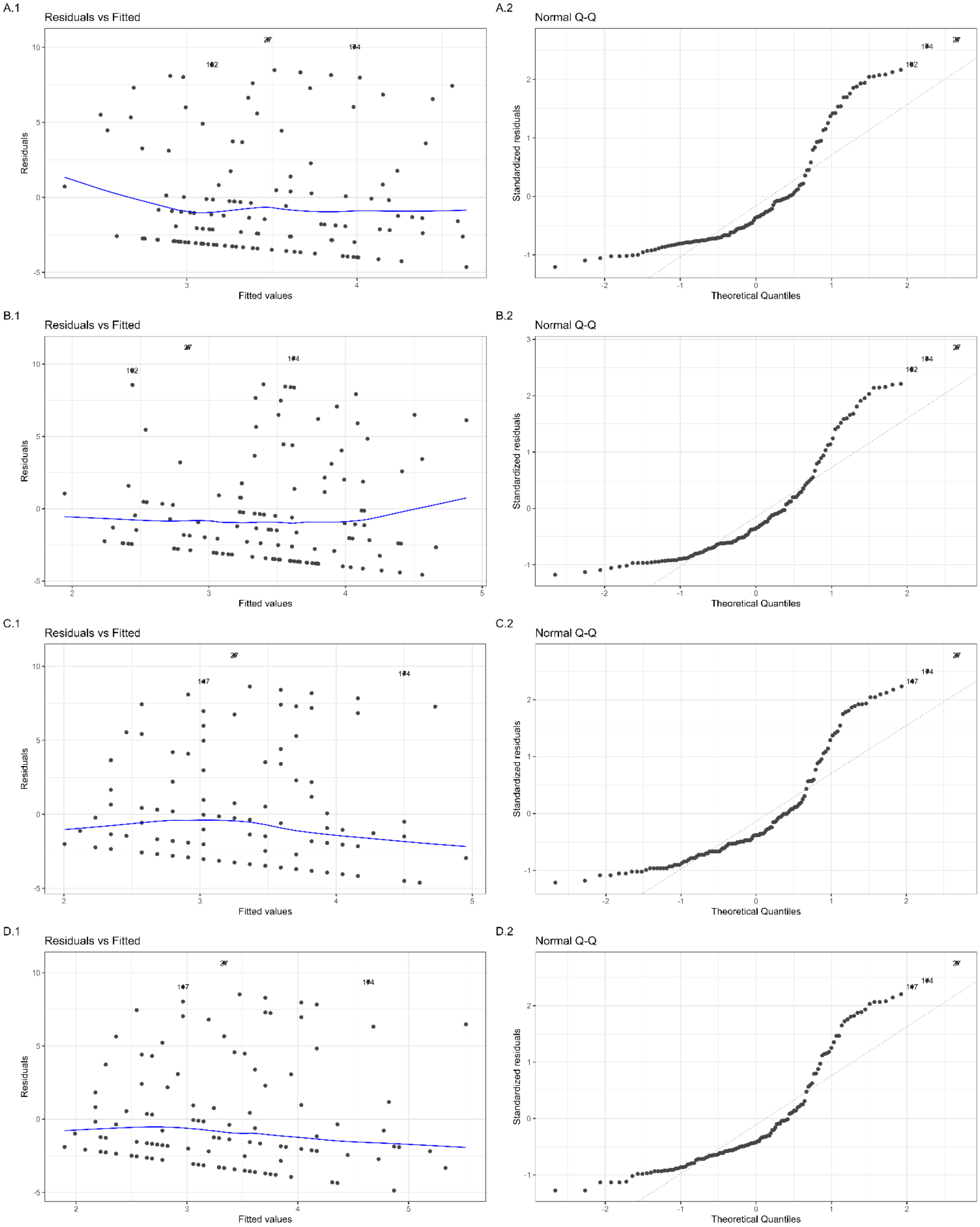

Supplement: sfae300_Supplemental_File [file sfae300_supplemental_file.pdf]
